# Assessing clinical investigators’ perceptions of relevance and competency of clinical trials skills: An international AIDS Malignancy Consortium (AMC) study

**DOI:** 10.1017/cts.2020.520

**Published:** 2020-08-07

**Authors:** Jeannette Y. Lee, Shelly V Lensing, Maria T. Botello-Harbaum, Rebecca Medina, Meredith Zozus

**Affiliations:** 1 University of Arkansas for Medical Sciences, Little Rock, AR, USA; 2 The Emmes Company, LLC, Rockville, MD, USA; 3 University of Texas Health Sciences Center, San Antonio, TX, USA

**Keywords:** HIV malignancies, core competency framework, training, clinical trial needs assessment, learning objectives

## Abstract

**Introduction:**

The AIDS Malignancy Consortium (AMC) conducts clinical trials of therapeutic and prevention strategies for cancer in people living with HIV. With its recent expansion to Sub-Saharan Africa and Latin America, there was a need to increase the competence of clinical investigators (CIs) to implement clinical trials in these regions.

**Methods:**

AMC CIs were invited to complete a survey to assess role-relevance and self-perceived competence based on the Joint Task Force for Clinical Trials Competency domains.

**Results:**

A total of 40 AMC CIs were invited to complete the questionnaire and 35 responded to the survey. The data management and informatics and engaging with communities’ domains were lowest in the average proportion of CIs rating themselves high (scores of 3–4) for self-perceived competency (46.6% and 44.2%) and role-relevance (61.6% and 67.5%), whereas, the ethical and participant safety considerations domain resulted in the highest score for competency (86.6%) and role-relevance (93.3%). In the scientific concepts and research design domain, a high proportion rated for competency in evaluating study designs and scientific literature (71.4% and 74.3%) but a low proportion for competency for designing trials and specimen collection protocols (51.4% and 54.3%).

**Conclusions:**

Given the complexity of AMC clinical research, these results provide evidence of the need to develop training for clinical research professionals across domains where self-perceived competence is low. This assessment will be used to tailor and prioritize the AMC Training Program in clinical trial development and management for AMC CIs.

## Introduction

Clinical trials are widely viewed as the “gold standard” since they are required *to prospectively evaluate the risks and benefits of a drug, device, behavioral intervention, or other forms of treatment* [1]. Over the years, there has been an increase in clinical trials conducted worldwide [2]. With the increase in the number of clinical trials that are developed and implemented, there is a commensurate demand for a workforce that can support these studies [3–5]. Conducting a clinical trial requires a team who collectively are familiar with regulatory requirements, reporting efficacy and safety measures, ethical considerations, data management, and analysis considerations [6]. There has been an increasing effort to establish and implement training requirements by several clinical research organizations [7, 8]. For studies conducted in the USA under the auspices of the federal government, clinical trial professionals are required, at a minimum, to maintain certification of training in human subjects’ research protections and good clinical practices (GCPs).

The Joint Task Force on Clinical Trial Competencies (JTF-CCT) established a core competency framework (CCF) to assess individuals’ role-relevance and self-competency for clinical research-related domains comprised of essential skills. The AIDS Malignancy Consortium (AMC) adopted the JTF-CCF to shape and enhance the professional development of their clinical trial workforce and to establish an online training program. The AMC was established in 1995 to prevent and treat cancer in HIV-infected persons by conducting clinical trials domestically and internationally in Sub-Saharan Africa (SSA) and in Latin America (LATAM). As the HIV epidemic has shifted to the developing world, the AMC has expanded its clinical trial activities to SSA in 2010 and to LATAM in 2018. This paper presents the results of the training needs assessment survey using the JTF-CCF survey to assess clinical investigators (CIs). The aim of this paper is to compare the scores for role-relevance and self-competence in order to identify where the greatest training needs are among the AMC CIs.

## Methods

### Survey and Study Design

The JTF-CCF assesses clinical professionals’ role-relevance and self-competency for skills falling under the following domains: (1) Scientific Concepts and Research Design, (2) Ethical and Participant Safety Considerations, (3) Medicines Development and Regulation, (4) Clinical Trials Operations, (5) Study and Site Management, (6) Data Management and Informatics, (7) Leadership and Professionalism, and (8) Communication and Teamwork. As advice is sought from community representatives to facilitate participant recruitment and retention, an additional domain was added to assess community engagement [9, 10]. The survey has been adapted by the Clinical and Translational Science Centers and customized to be administered to CIs, study coordinators, and data managers. The Hennessy–Hicks training needs analysis [11] was followed to identify training needs and to prioritize training for AMC CIs. The role-relevance rating assesses how important a task is to the respondent’s job, whereas the self-competence rating measures how well a task is currently performed. The bigger the difference between the relevance and the competence scores, the greater the training need.

The AMC Training Needs Assessment was administered through the online SurveyMonkey™ platform. It was launched on September 14, 2018 for sites in SSA and on September 21, 2018 for the sites in LATAM; both surveys were closed on October 19, 2018. The assessment was translated from English to Spanish and Portuguese and it included two demographic questions, AMC site name and country, and 52 items related to research activities (refer to Supplementary Material). AMC CIs were asked to rate their role-relevance and competence within a domain using a five-point scale (0–4). The respective anchor question and the response options are shown in Table 1.

**Table 1.** Self-perceived role-relevance and self-competence response options

Questions	Response options
**Role-relevance**	
*How important is the activity to the successful performance of your role?*	0 Unnecessary, no relevance to my role 1 Has some relevance to my role, but not my responsibility 2 Relevant to my role, but not a major component 3 Significant to my role and part of my job responsibilities 4 Major part of my responsibility or supervisory expectations
**Competence**	
*How well do you consider that you currently perform this activity?*	0 Never been exposed to this content 1 Aware of the content, but never needed to become further informed 2 Exposed and sufficiently aware of content that I can look up what might be necessary for my role 3 Competent – able to interpret or discuss concepts and use knowledge to solve simple problems based on application concepts 4 Mastery – able to apply knowledge to complex problems, integrate information, and create solutions

### Data Analysis

The self-competence level and role-relevance scores were averaged across skills within a given domain and graphed. The proportion of respondents who considered a skill relevant to their role was estimated as the proportion whose responses were 3 or 4. Similarly, the proportion of respondents who considered themselves competent with respect to a skill was estimated as the proportion whose responses were 3 or 4. Within each domain, the mean proportions of investigators who rated the skill as relevant to their role in conducting clinical trials and who considered themselves competent were calculated. Consistent with Sonstein et al. [8], in the present study, an average score of 60% or more implies “more competent” or “more relevant,” and a mean value of <60% implied “less competent” or “less relevant.” To determine the AMC training needs and the course development priorities, the mean self-competence score on the original 0–4 scale was subtracted from the mean role-relevance score [11]. The data were analyzed using SAS (version 9.4).

## Results

Investigators from 11 AMC sites were invited to participate; 8 sites were in SSA countries and 4 in countries in LATAM (data not shown). A total of 40 AMC CIs were invited to participate in the training needs assessment; of those, 35 submitted their responses for an 87.5% response rate. There were 13 CIs from LATAM countries and 22 from SSA countries (Table 2).

**Table 2.** AIDS Malignancy Consortium (AMC) clinical investigators (CIs) by country (*n* = 35)

Country	*n*	%
**LATAM (** ***n*** ** = 13)**		
Argentina	5	38.5
Brazil	7	53.8
Mexico	1	7.7
**SSA (** ***n*** ** = 22)**		
Kenya	2	9.1
Malawi	3	13.6
South Africa	9	40.9
Tanzania	4	18.2
Zimbabwe	4	18.2

**Fig. 1.**
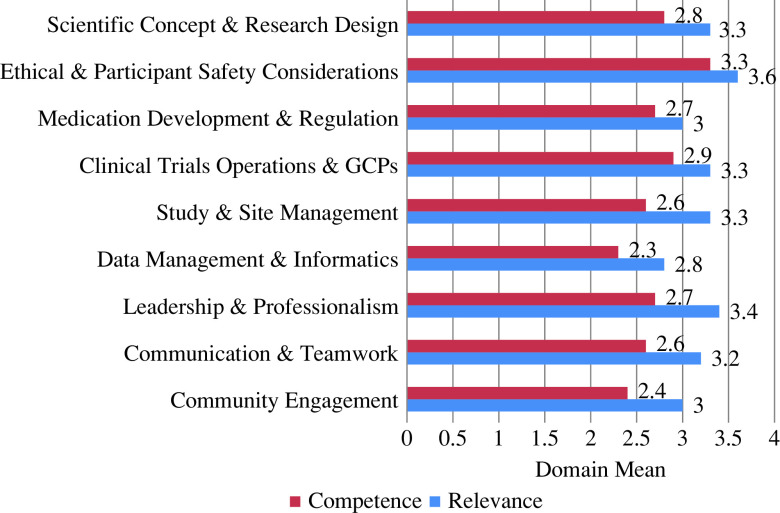
Domain means for relevance to role and competency. Scores for individual items (skills) were averaged within each domain. GCP, good clinical practices.

Figure 1 shows the mean domain score for relevance and competence as self-rated by CIs. CIs perceived themselves as highly competent in their knowledge of factors related to ethics and participant considerations. The data management and informatics and the community engagement domains had lower scores for competency domains. The results of the study showed that the greatest difference between the role-relevance and competence ratings were in the following domains: study and site management (difference of 0.7), leadership and professionalism (difference of 0.7), communication and teamwork (difference of 0.6), community engagement (difference of 0.6), and scientific concept and research design (difference of 0.5).

### Perceptions of Competence and Relevance

Table 3 presents the average percentage of CIs who rated their skills high (score of 3 or 4) within each research domain for competence and relevance. The ethical and participant safety considerations had the highest mean role-relevance and self-competence percentages with 93.3% and 86.6%, respectively. The perceived role-relevance was high in the clinical trials operations and GCPs domain with a mean of 87%, followed by leadership and professionalism (87.5%). The only skill with less than 60% on the role-relevance scale was to summarize the process of electronic data capture and the importance of information technology in data collection, capture and management with 50% rating this skill high.

**Table 3.** Clinical research domains among AIDS Malignancy Consortium (AMC) clinical investigators (CIs)

Clinical research domains	Role-relevance (%)	Self-competence (%)
**Scientific concept and research design**		
Identify clinically important questions that are potentially testable clinical research hypotheses, through review of the professional literature	91.4	74.3
Evaluate the appropriateness, advantages, and disadvantages of clinical trial designs	91.4	71.4
Design a clinical trial that operationalizes a testable hypothesis	80.0	51.4
Implement operational adjustments in clinical trials needed for HIV+ populations	88.6	65.7
Design biospecimen collection processes to address the protocol objective appropriate for people living with HIV	77.1	54.3
Critically evaluate results from clinical trials	91.2	82.9
Domain mean	86.6	66.7
**Ethical and participant safety considerations**		
Differentiate between standard of care and clinical trial activities	97.1	91.2
Define the concepts “clinical equipoise” as related to the conduct of a clinical trial	88.6	74.3
Apply relevant principles of human subject protections and privacy throughout all stages of a clinical trial	100.0	97.1
Define vulnerable populations and additional safeguards needed for the protection of those populations	94.3	88.6
Explain how inclusion and exclusion criteria are included in a clinical trial protocol to assure human subject protection	91.4	91.4
Summarize the principles and methods of distribution and balancing risk and benefit throughout the selection and management of clinical trial subjects	88.6	77.1
Domain mean	93.3	86.6
**Medication development and regulation**		
Develop specific processes and phases that must be followed to satisfy regulatory requirements	77.1	60.0
Identify my country’s regulatory agencies and the role of the agency in clinical trial oversight	74.3	60.0
Explain the safety reporting requirement of regulatory agencies	74.3	57.1
Differentiate the roles and responsibilities of the sponsor, investigator, and supporting study team for investigational product development	80.0	60.0
Domain mean	76.4	59.3
**Clinical trials operations and good clinical practices (GCPS)**		
Explain how the design, purpose, and conduct of individual clinical trials fit into the goal of achieving a new intervention	91.4	71.4
Understand the purpose of a Clinical Research Organization (CRO) and the role of the CRO in the clinical trial	77.1	62.9
Describe the roles and responsibilities of the clinical investigational team when conducting a clinical trial	88.6	80.0
Identify my site’s stakeholders	94.3	74.3
Evaluate the conduct and documentation of clinical trials as required for compliance with GCP guidelines	88.6	80.0
Describe appropriate control, storage, and dispensing of investigational products	74.3	57.1
Differentiate and identify serious and nonserious adverse events	97.1	88.6
Describe the role of the investigator in reviewing and assessing each adverse event	94.3	85.7
Describe the serious adverse event/adverse event reporting requirements to institutional review boards, sponsors, and regulatory authorities	94.3	80.0
Categorize adverse events with the standard controlled terminology such as the common terminology criteria for adverse events	91.4	71.4
Describe the purpose and process for monitoring clinical trials	80.0	67.7
Host a clinical trial audit and respond to audit findings	74.3	51.4
Follow regulatory reporting processes for unanticipated adverse events during a clinical trial	85.7	62.9
Domain mean	87.0	71.8
**Study and site management**		
Evaluate proposed clinical trials for feasibility and scope, given available time and resources	91.2	73.5
Develop and manage the financial, timeline, and cross-disciplinary personnel resources necessary to conduct a clinical trial	82.4	50.0
Evaluate clinical trial risk and determine training to mitigate risk and improve study quality in the context of applicable regulations	82.4	58.8
Develop strategies to manage participant recruitment, study activities, and track progress	88.2	67.7
Identify the legal and regulatory responsibilities, liabilities, and accountabilities that are involved in the conduct of clinical trials	79.4	50.0
Identify and explain the specific procedural, documentation, and oversight requirements of PIs, sponsors, and regulatory authorities related to the conduct of the clinical trial	82.4	55.9
Domain mean	84.3	59.3
**Data management and informatics**		
Describe the role of statistics and informatics	61.8	50.0
Describe the flow and management of data through a clinical trial	67.7	55.9
Identify best practices for data standardization, collection, and capture and management for a clinical trial	60.6	44.1
Summarize the process of electronic data capture and the importance of information technology in data collection, capture and management	50.0	35.3
Describe the GCP requirements for data correction and queries	67.7	55.9
Describe the significance of data quality assurance systems and how standard operating procedures are used to guide these processes	61.8	44.1
Describe the requirements and local procedures for archiving study records	61.8	41.2
Domain mean	61.6	46.6
**Leadership and professionalism**		
Apply the principles and practices of leadership in management and mentorship	85.3	61.8
Identify, analyze, and address ethical and professional conflicts associated with the conduct of clinical trials	94.1	61.8
Identify and apply professional guidelines and codes of ethics as they related to the conduct of clinical trials	85.3	67.7
Recognize the potential effects of cultural diversity and the need for cultural competency in the design and conduct of clinical trials	85.3	58.8
Domain mean	87.5	62.5
**Communication and teamwork**		
Discuss the relationship and appropriate communication between the sponsor, contract research organizations, and clinical research site	79.4	55.9
Write a scientific publication reporting the results of a clinical trial	82.4	55.9
Effectively communicate the content and relevance of clinical trial findings to colleagues, advocacy groups, and the nonscientist community	73.5	64.7
Describe the methods necessary to work effectively with multidisciplinary and interprofessional research teams	85.3	64.7
Domain mean	80.2	60.3
**Engaging with communities**		
Form and maintain equitable partnerships with public health departments, local agencies, and community organizations to understand local population health needs and to jointly address them through clinical research	67.7	47.1
Form and/or maintain interactions with local Community Advisory Board (CAB) to inform and educate members regarding clinical studies and in turn to receive feedback from CAB members regarding all aspects of clinical trials	67.7	41.2
Domain mean	67.7	44.2

The domains with competency levels of 60% or less were medication development and regulation (59.3%), study and site management (59.3%), data management and informatics (46.6%), and community engagement (44.2%). These domains also showed a low role-relevance score on all their respective skills. In the scientific concept and design domain, investigators demonstrated greater ability to evaluate clinical trial designs or results (82.9%), but less confidence in their ability to design a clinical trial that operationalizes a testable hypothesis (51.4%).

In the clinical trials and GCPs domain, CIs expressed a high competence in their ability to review, assess, and report adverse events (80%), but felt less able to describe the appropriate control, storage, and dispensing of investigational products (57.1%) and host a clinical trial audit and respond to audit findings (51.4%). With respect to the study and site management domain, investigators’ competence was high for feasibility evaluation for trials and for managing recruitment and study progress (73.5%), but less confident in their abilities to manage resources and legal and regulatory responsibilities (50%).

## Discussion

This paper aimed to assess role-relevance and self-perceived competence across the JTF-CC domains and identify where the greatest training needs are among the AMC CIs. As recommended by Kilic and collaborators [12], training needs should be identified before the development of training programs. The goal of the AMC Training Program (ATP) is to improve and enhance clinical staff sites’ performance, while at the same time standardizing the process and consistency in the delivery of training. The results of the training needs assessment show that AMC CIs rated themselves as competent in their ability to review clinical trial design and results but were less confident in their ability to design a study. The JTF-CCT administered a survey similar to the one we used in this study among a multinational group of clinical research professionals that included investigators and clinical research associates or coordinators [8]. The findings from the JTF-CCT showed were consistent with our results, in which respondents indicated being competent, with a mean value of 60% or higher, in the ethical and participant safety considerations and the clinical trials operations. Within the scientific concept and research design domain, AMC CIs rated the design of a clinical trial that operationalizes a testable hypothesis as another competency where additional training was indicated. This finding was not consistent with the work of Barratt and Fulop [13] among allied health professionals, managers, and nurses, who indicated that designing research studies as one of the least important domains. Our data also show that CIs considered that they are competent in the medications and regulations domain and training in this area might not be a priority. Imamura et al. [14] reported that medications and regulation were among the lowest competencies in regard to relevance and self-competence. Only 28% found this domain to be significant to their position and 24% felt competent in this domain.

AMC CIs rated the data management and informatics domain low for most of the skills or competencies for that domain, suggesting that training in this area could be considered given the low competency. However, the role-relevance was also rated low. This may be attributable for CI reliance on informatics professionals to design and operationalize data collection and handling for a study but may also be attributable to lack of awareness or misconception of data-related tasks as clerical in nature. This was not explored within the survey. Our findings conflict with the results of a training needs assessment survey of faculty members and students of the three minority medical institutions that make up the Puerto Rico Clinical Translational Research Consortium (PRCTRC); faculty members ranked statistical and informatics as one of the high priority areas for training based on high relevance and low competency [15].

The community engagement domain is new to our survey, but the findings suggest that this is an area for training for our investigators. It is likely that enhancing skills in this area may need to be tailored to a specific geographic region or clinic and reflect cultural norms. The need for development in the community engagement domain reflects the fact that this area has not historically been covered in education in clinical trials and has not been considered one of the core competencies [7, 8]. Increasing familiarity and competency in this domain as community engagement has been shown to be useful in the design and implementation of cancer clinical trials [16] and enhancing the participation of underrepresented minorities [17, 18].

In this study, the leadership and professionalism competence levels were lower than those reported by Sonstein [8]. This may reflect a difference in the experience level of those holding leadership positions in clinical trials between the two studies. Most of the AMC investigators are relatively recent additions to the multicenter clinical trial network and many are beginning to assume leadership positions within the organization so these competence levels are expected to rise.

The Communication and Teamwork domain encompasses communication within the study team with outside stakeholders as well as communicating scientific results. Since the AMC is a multicenter and multinational clinical trial group, the complexity and extent of the needs for communication and teamwork are greater than that for single-center clinical trials.

The AMC CIs reported a high level of competence in managing patient participation in clinical trials in terms of recruitment and retention, and the assessment and reporting of adverse events. They felt less comfortable with managing resources for a clinical trial and site monitoring. These aspects of clinical trials require accessing site-level resources to support financial, legal, and regulatory needs.

This training needs assessment survey has been extremely informative in identifying the areas for professional development that will facilitate clinical trial workforce development at the AMC sites in Sub-Saharan Africa and LATAM. The AMC is working to incorporate professional development offerings to our investigators using an online learning management system. A general curriculum has been developed and it is now being tailored to the needs of the AMC’s clinic staff. Additionally, the AMC-specific curriculum has prioritized the release of courses, given the findings of this questionnaire.

Overall, CIs recognized the relevance of the core competencies required for conducting clinical trials, but there was variability in their self-perceived abilities to apply the competencies. Based on the self-reported competencies, a series of training and professional development modules are under development. These results will facilitate the prioritization of domains for training as the AMC expands its activities internationally and reduce any gaps in the provision of training.

## Supplementary material

For supplementary material accompanying this paper visit https://doi.org/10.1017/cts.2020.520. click here to view supplementary material
