# Supplementary material for: Assessing clinical investigators’ perceptions of relevance and competency of clinical trials skills: An international AIDS Malignancy Consortium (AMC) study
Source: J Clin Transl Sci. 2020 Aug 7;5(1):e28. doi: 10.1017/cts.2020.520 (PMC8057474; doi:10.1017/cts.2020.520)
Supplement: Supplementary file 1 [file S2059866120005208sup001.docx]

**Appendix 1.**

**AMC Training Needs Assessment**

**Clinical Investigator Survey**

Objective: The purpose of this survey is to identify areas in which the AIDS Malignancy Consortium Operations (AMC) can provide additional training.

Investigators responsible for AMC studies or who are expected to contribute to the scientific agenda are required to complete this survey. Your participation will allow the AMC to develop a more effective training curriculum for the Group.

All responses to this survey are anonymous, the answers you provide cannot be linked back to you. Responses will be reviewed and assessed in aggregate.

**Site:**

- Bugando Medical Centre
- Fundación Huesped
- Hospital Universitario Professor Edgar Santos (HUPES)
- Instituto Nacional de Cancerologia
- Instituto Nacional de Cancer Jose Alencar (INCA)
- Kamuzu Central Hospital
- Moi Teaching and Referral Hospital
- Parirenyatwa Hospital
- Stellenbosch University
- Uganda Cancer Institute
- University of Witwatersrand
- University of Zimbabwe, College of Health Sciences

**Country:**

- Argentina
- Brazil
- Kenya
- Malawi
- Mexico
- South Africa
- Tanzania
- Uganda
- Zimbabwe

**___________________________________________________________________**

Instructions for Investigators

In order to perform effectively as a clinical site investigator or PI of multi-center clinical trials you need relevant skills. You will see listed below a range of skilled activities commonly performed by AMC clinical site investigators or PIs of multi-center clinical trials.

Look at each of these competencies and rate each one by indicating:

(1) How ***important*** the activity is to the successful performance of your role.
0- Unnecessary, no relevance to my role
1- Has some relevance to my role, but not my responsibility
2- Relevant to my role, but not a major component
3- Significant to my role and part of my job responsibilities
4- Major part of my responsibility or supervisory expectations

(2) How well you ***perform*** that activity today.

0- Never been exposed to this content
1- Aware of the content, but never needed to become further informed
2- Exposed and sufficiently aware of content that I can look up what might be necessary for my role
3- Competent – Able to interpret or discuss concepts and use knowledge to solve simple problems based on application concepts
4- Mastery – Able to apply knowledge to complex problems, integrate information, and create solutions

**SCIENTIFIC CONCEPTS AND RESEARCH DESIGN**

1. **Identify clinically important questions that are potentially testable clinical research hypotheses, through review of the professional literature.**

- How important is this activity to the successful performance of your role?
- How well do you consider that you currently perform this activity?

1. **Evaluate the appropriateness, advantages and disadvantages of clinical trial designs.**

- How important is this activity to the successful performance of your role?
- How well do you consider that you currently perform this activity?

1. **Design a clinical trial that operationalizes a testable hypothesis.**

- How important is this activity to the successful performance of your role?
- How well do you consider that you currently perform this activity?

1. **Implement operational adjustments in clinical trials needed for HIV+ populations.**

- How important is this activity to the successful performance of your role?
- How well do you consider that you currently perform this activity?

1. **Design biospecimen collection processes to address the protocol objectives appropriate for people living with HIV (for example, quantity/volume of specimen, and subject informed consent).**

- How important is this activity to the successful performance of your role?
- How well do you consider that you currently perform this activity?

1. **Critically evaluate results from clinical trials.**
   - How important is this activity to the successful performance of your role?
   - How well do you consider that you currently perform this activity?

**ETHICAL AND PARTICIPANT SAFETY CONSIDERATIONS**

1. **Differentiate between standard of care and clinical trial activities.**

- How important is this activity to the successful performance of your role?)
- How well do you consider that you currently perform this activity?

1. **Define the concepts “clinical equipoise” as related to the conduct of a clinical trial.**

- How important is this activity to the successful performance of your role?
- How well do you consider that you currently perform this activity?

1. **Apply relevant principles of human subject protections and privacy throughout all stages of a clinical trial.**

- How important is this activity to the successful performance of your role?
- How well do you consider that you currently perform this activity?

1. **Define vulnerable populations and additional safeguards needed for protection of those populations.**

- How important is this activity to the successful performance of your role?
- How well do you consider that you currently perform this activity?

1. **Explain how inclusion and exclusion criteria are included in a clinical trial protocol to assure human subject protection.**

- How important is this activity to the successful performance of your role?
- How well do you consider that you currently perform this activity?

1. **Summarize the principles and methods of distributing and balancing risk and benefit through selection and management of clinical trial subjects.**

- How important is this activity to the successful performance of your role?
- How well do you consider that you currently perform this activity?

**MEDICATION DEVELOPMENT AND REGULATION**

**In the context of conducting clinical trials answer the following survey items:**

1. **Describe specific processes and phases that must be followed to satisfy regulatory requirements.**

- How important is this activity to the successful performance of your role?
- How well do you consider that you currently perform this activity?

1. **Identify my country’s regulatory agencies and the role of the agency in clinical trial oversight**

- How important is this activity to the successful performance of your role?
- How well do you consider that you currently perform this activity?

1. **Explain the safety reporting requirements of regulatory agencies.**

- How important is this activity to the successful performance of your role?
- How well do you consider that you currently perform this activity?

1. **Differentiate the roles and responsibilities of the sponsor, investigator, and supporting study team for investigational product development.**

- How important is this activity to the successful performance of your role?
- How well do you consider that you currently perform this activity?

**CLINICAL TRIALS OPERATIONS AND GOOD CLINICAL PRACTICE (GCPs)**

1. **Explain how the design, purpose, and conduct of individual clinical trials fit into the goal of achieving a new intervention.**

- How important is this activity to the successful performance of your role?
- How well do you consider that you currently perform this activity?

1. **Understand the purpose of a Clinical Research Organization (CRO) and the role of the CRO in the clinical trial.**

- How important is this activity to the successful performance of your role?
- How well do you consider that you currently perform this activity?

1. **Describe the roles and responsibilities of the clinical investigational team when conducting a clinical trial.**

- How important is this activity to the successful performance of your role?
- How well do you consider that you currently perform this activity?

1. **Identify my site’s stakeholders (signing authority, finance, Institutional Review Board, Community Advisory Board, etc.)**

- How important is this activity to the successful performance of your role?
- How well do you consider that you currently perform this activity?

1. **Evaluate the conduct and documentation of clinical trials as required for compliance with Good Clinical Practice (GCP) guidelines.**

- How important is this activity to the successful performance of your role?
- How well do you consider that you currently perform this activity?

1. **Describe appropriate control, storage, and dispensing of investigational products.**

- How important is this activity to the successful performance of your role?
- How well do you consider that you currently perform this activity?

1. **Differentiate and identify serious and non-serious adverse events (AEs).**

- How important is this activity to the successful performance of your role?
- How well do you consider that you currently perform this activity?

1. **Describe the role of the investigator in reviewing and assessing each adverse event.**
   - How important is this activity to the successful performance of your role?
   - How well do you consider that you currently perform this activity?
2. **Describe the Serious Adverse Events (SAE)/Adverse Events (AE) reporting requirements to Institutional Review Boards) IRBs/ Institutional Ethics Committees (IECs), sponsors, and regulatory authorities.**

- How important is this activity to the successful performance of your role?
- How well do you consider that you currently perform this activity?

1. **Categorize adverse events with standard controlled terminology such as Common Terminology Criteria for Adverse Events (CTCAE)**

- How important is this activity to the successful performance of your job?
- How well do you consider that you currently perform this activity?

1. **Describe the purpose and process for monitoring clinical trials.**

- How important is this activity to the successful performance of your role?
- How well do you consider that you currently perform this activity?

1. **Host a clinical trial audit and respond to audit findings.**

- How important is this activity to the successful performance of your role?
- How well do you consider that you currently perform this activity?

1. **Follow regulatory reporting processes for unanticipated adverse events during a clinical trial.**

- How important is this activity to the successful performance of your role?
- How well do you consider that you currently perform this activity?

**STUDY AND SITE MANAGEMENT**

1. **Evaluate proposed clinical trials for feasibility and scope, given available time and resources.**

- How important is this activity to the successful performance of your role?
- How well do you consider that you currently perform this activity?

1. **Develop and manage the financial, timeline, and cross-disciplinary personnel resources necessary to conduct a clinical trial.**

- How important is this activity to the successful performance of your role?
- How well do you consider that you currently perform this activity?

1. **Evaluate clinical trial risk and determine training to mitigate risk and improve study quality in the context of applicable regulations.**

- How important is this activity to the successful performance of your role?
- How well do you consider that you currently perform this activity?

1. **Develop strategies to manage participant recruitment, study activities, and track progress.**

- How important is this activity to the successful performance of your role?
- How well do you consider that you currently perform this activity?

1. **Identify the legal and regulatory responsibilities, liabilities, and accountabilities that are involved in the conduct of clinical trials.**

- How important is this activity to the successful performance of your role?
- How well do you consider that you currently perform this activity?

1. **Identify and explain the specific procedural, documentation, and oversight requirements of PIs, sponsors and regulatory authorities related to the conduct of a clinical trial.**

- How important is this activity to the successful performance of your role?
- How well do you consider that you currently perform this activity?

**DATA MANAGEMENT AND INFORMATICS**

**36. Describe the role of statistics and informatics.**

- How important is this activity to the successful performance of your role?
- How well do you consider that you currently perform this activity?

**37. Describe the flow and management of data through a clinical trial.**

- How important is this activity to the successful performance of your role?
- How well do you consider that you currently perform this activity?

1. **Identify best practices for data standardization, collection, capture, and management for a clinical trial.**

- How important is this activity to the successful performance of your role?
- How well do you consider that you currently perform this activity?

1. **Summarize the process of electronic data capture and the importance of information technology in data collection, capture and management.**

- How important is this activity to the successful performance of your role?
- How well do you consider that you currently perform this activity?

1. **Describe the Good Clinical Practice (GCP) requirements for data correction and queries.**

- How important is this activity to the successful performance of your role?
- How well do you consider that you currently perform this activity?

1. **Describe the significance of data quality assurance systems and how standard operating procedures are used to guide these processes.**

- How important is this activity to the successful performance of your role?
- How well do you consider that you currently perform this activity?

1. **Describe the requirements and local procedures for archiving study records.**

- How important is this activity to the successful performance of your job?
- How well do you consider that you currently perform this activity?

**LEADERSHIP AND PROFESSIONALISM**

1. **Apply the principles and practices of leadership in management and mentorship.**

- How important is this activity to the successful performance of your role?
- How well do you consider that you currently perform this activity?

1. **Identify, analyze, and address ethical and professional conflicts associated with the conduct of clinical trials.**

- How important is this activity to the successful performance of your role?
- How well do you consider that you currently perform this activity?

1. **Identify and apply professional guidelines and codes of ethics as they relate to the conduct of clinical trials.**

- How important is this activity to the successful performance of your role?
- How well do you consider that you currently perform this activity?

1. **Recognize the potential effects of cultural diversity and the need for cultural competency in the design and conduct of clinical trials.**

- How important is this activity to the successful performance of your role?
- How well do you consider that you currently perform this activity?

**COMMUNICATION AND TEAMWORK**

1. **Discuss the relationship and appropriate communication between sponsor, contract research organizations, and clinical research site.**

- How important is this activity to the successful performance of your role?
- How well do you consider that you currently perform this activity?

1. **Write a scientific publication reporting the results of a clinical trial.**

- How important is this activity to the successful performance of your role?
- How well do you consider that you currently perform this activity?

1. **Effectively communicate the content and relevance of clinical trial findings to colleagues, advocacy groups, and the nonscientist community.**

- How important is this activity to the successful performance of your role?
- How well do you consider that you currently perform this activity?

1. **Describe the methods necessary to work effectively with multidisciplinary and interprofessional research teams.**

- How important is this activity to the successful performance of your role?
- How well do you consider that you currently perform this activity?

**ENGAGING WITH COMMUNITIES**

1. **Form and maintain equitable partnerships with public health departments, local agencies, and community organizations, to understand local population health needs and to jointly address them through clinical research.**

- How important is this activity to the successful performance of your ***desired*** role?
- How well do you consider that you currently perform this activity?

1. **Form and/or maintain interactions with local Community Advisory Board (CAB) to inform and educate members regarding clinical studies and in turn to receive feedback from CAB members regarding all aspects of clinical trials.**

- How important is this activity to the successful performance of your ***desired*** role?
- How well do you consider that you currently perform this activity?
